# Highly efficient multiplex base editing: One-shot deactivation of eight genes in *Shewanella oneidensis* MR-1

**DOI:** 10.1016/j.synbio.2022.09.005

**Published:** 2022-10-13

**Authors:** Yaru Chen, Meijie Cheng, Yan Li, Lin Wang, Lixia Fang, Yingxiu Cao, Hao Song

**Affiliations:** a Frontier Science Center for Synthetic Biology and Key Laboratory of Systems Bioengineering (Ministry of Education), School of Chemical Engineering and Technology, Tianjin University, Tianjin, 300072, China; b Key Laboratory of Systems Bioengineering (Ministry of Education), Tianjin University, Tianjin, 300072, China

**Keywords:** Multiplex gene editing, Electroactive microorganisms, Base editing, Multiplexed engineering, CRISPR, Extracellular electron transfer

## Abstract

Obtaining electroactive microbes capable of efficient extracellular electron transfer is a large undertaking for the scalability of bio-electrochemical systems. Inevitably, researchers need to pursue the co-modification of multiple genes rather than expecting that modification of a single gene would make a significant contribution to improving extracellular electron transfer rates. Base editing has enabled highly-efficient gene deactivation in model electroactive microbe *Shewanella oneidensis* MR-1. Since multiplexed application of base editing is still limited by its low throughput procedure, we thus here develop a rapid and efficient multiplex base editing system in *S. oneidensis*. Four approaches to express multiple gRNAs were assessed firstly, and transcription of each gRNA cassette into a monocistronic unit was validated as a more favorable option than transcription of multiple gRNAs into a polycistronic cluster. Then, a smart scheme was designed to deliver one-pot assembly of multiple gRNAs. 3, 5, and 8 genes were deactivated using this system with editing efficiency of 83.3%, 100% and 12.5%, respectively. To offer some nonrepetitive components as alternatives genetic parts of sgRNA cassette, different promoters, handles, and terminators were screened. This multiplex base editing tool was finally adopted to simultaneously deactivate eight genes that were identified as significantly downregulated targets in transcriptome analysis of riboflavin-overproducing strain and control strain. The maximum power density of the multiplex engineered strain HRF(8BE) in microbial fuel cells was 1108.1 mW/m^2^, which was 21.67 times higher than that of the wild-type strain. This highly efficient multiplexed base editing tool elevates our ability of genome manipulation and combinatorial engineering in *Shewanella*, and may provide valuable insights in fundamental and applied research of extracellular electron transfer.

## 1 Introduction

Extracellular electron transfer (EET) efficiency of electroactive microbes (EAMs) is of paramount importance to the feasibility of bio-electrochemical systems (BES) [1], for instance, electricity-production microbial fuel cells (MFCs) [2], chemical-production microbial electrosynthesis (MES) [3], H_2_-production microbial electrolysis cells (MEC) [4], biotoxicity-detection electrochemical microbial biosensors [5], seawater-desalination microbial desalination cells [6]. An increasing focus on sustainability has led to an ongoing global effort to understand and engineer model EAM, *Shewanella oneidensis* MR-1 [2]. Given our limited knowledge on the complex relation between cellular processes and the efficiency of EET, it is highly desirable to investigate more gene targets and reveal the association between genotype and phenotype [7]. To this end, it is inevitable that multiple genes need to be modified, especially for those interacting synergistically in the electron transfer process [8].

Various tools derived from Clustered Regularly Interspaced Short Palindromic Repeats (CRISPR) have been widely used for genetic manipulation of *S. oneidensis*. Gene regulation tools include CRISPR interference (CRISPRi) for gene downregulation [9], CRISPR activation (CRISPRa) for gene upregulation [10], and CRISPR-PAIR for multi-mode regulation [11]. Meanwhile, gene editing tools have realized plenty of functions such as gene deactivation [12], gene knockout [13], gene replacement [14], gene insertion [15], and insertion of large fragments [16]. Amidst these technologies, base editing has been generally acknowledged as an efficacious way to deactivate genes, circumventing user-unfriendly gene knockout with introduction of DNA double-strand breaks and multiple components (e.g., ssDNA repair template) [17]. Base editor mediates C to T conversion in the CAG, CAA, CGA, TGG codons to generate premature stop codons (TAA, TAG and TGA) [18]. It has been demonstrated as a genome-level approach in genetic engineering and gene function identification [19,20]. Despite its conducive features, multiplexed application of base editing in *S. oneidensis* is still impeded by the low-throughput procedures of the current system [21]. As far as we know, base editors can edit at most two targets in a round for *S. oneidensis*, and to edit more than two loci, iterative methods must be used. Indeed, no method has so far been able to silence more than two discrete genes in a “one-shot” manner in *S. oneidensis*. The necessity of repetitive manipulation thus hinders the process of establishing complex regulatory networks and metabolic pathways at the underlying level. The disagreement between the absence of multiplexed base editing tools and the need of deciphering more gene connections makes it urgent to develop such strategies to perform extensive EET studies in a high-throughput manner.

The mainstay to developing a multiplex base editing system lies in the amounts and throughput of gRNAs, depending on the method of expressing multiple gRNAs, of which there are generally two types [22]. One method is to transcribe each gRNA cassette with individual promoter as a monocistronic unit [23,24]. The other is to transcribe all gRNAs into a polycistronic cluster using a single promoter, which is then processed via different avenues to release individual gRNAs [[25], [26], [27], [28], [29]]. Each gRNA needs to be flanked by cleavable RNA sequences, such as self-cleavable ribozyme sequences (e.g., hepatitis delta virus ribozyme) [28,30], exogenous cleavage protein recognition sequences (e.g., Cys4 recognition sequences) [25] and endogenous RNA processing sequences (e.g., tRNA sequences) [26]. It has remained unknown which method is more suitable for the existing base editing system of *S. oneidensis*.

Another prevailing challenge is the compact repetitive DNA sequences. Multiplexed systems always contain several long DNA repeats in both the sgRNAs and the genetic parts used to express them [31,32]. Active homologous recombination would result in the loss of gRNAs through spontaneous deletion, both in *E. coli* (for plasmid building) and *S. oneidensis*, triggering two issues: difficulty to assemble and genetic instability [33,34]. Avoidance of repetitive DNA sequences has been demonstrated to be helpful in *E. coli*, where simultaneous transcriptional repression of 22 genes was achieved by designing sgRNA expression cassettes consisting of highly nonrepetitive genetic parts [24]. Such pioneering work has led us to place great emphasis on exploring a number of available genetic elements for multiplexed base editing in *S. oneidensis*.

**Fig. 1:**
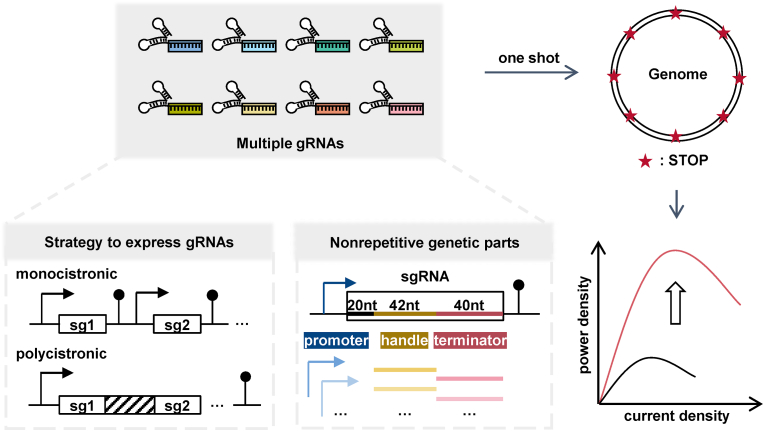
Highly efficient multiplex base editing system to enhance the EET efficiency in *Shewanella oneidensis*. Different strategies were evaluated to express multiple gRNAs, including monocistronic and polycistronic approaches. A number of nonrepetitive components, including promoters, handles, and terminators, were screened as alternatives genetic parts of the sgRNA cassette. This multiplex base editing tool allows for the simultaneous deactivation of 8 genes, elevating our ability for genome manipulation and combinatorial engineering of *Shewanella*, thus providing additional opportunities to improve the EET efficiency.

Here we represented a smart-assembly multiplex base editing tool for rapid genome engineering in *S. oneidensis* MR-1 (Fig. 1). Firstly, the appropriate method was evaluated to express multiple gRNAs. Each gRNA cassette transcribed as a monocistronic unit was validated as the more favorable approach than multiple gRNAs transcribed as a polycistronic cluster. Secondly, we designed a smart scheme to implement one-pot assembly of multiple gRNAs. 3, 5, and 8 genes were edited and the editing efficiency were 83.3%, 100%, and 12.5%, respectively. Thirdly, highly nonrepetitive genetic parts of sgRNA cassette, including promoters, handles, and terminators were screened to offer some viable components for further optimization. Lastly, this multiplex base editing tool was harnessed to deactivate 8 genes simultaneously, which were identified as the significantly downregulated targets in the transcriptome analysis of the riboflavin (RF)-overproducing strain and the control strain. The multiplex engineered strain HRF(8BE) achieved the maximum power density of 1108.1 mW/m^2^, 21.67 folds of the WT (wild-type *S. oneidensis* MR-1) strain. Collectively, this highly efficient multiplexed base editing tool sets the stage for genome combinatorial engineering during the course of BES advancement.

## 2 Materials and methods

### 2.1 Bacterial strains and culture conditions

The strains used in this study are listed in Table S4. *E. coli* DH5α and Trans1-T1 were used as general cloning strains, which should be cultivated aerobically at 37 °C in Luria–Bertani (LB) broth. *E. coli* WM3064 was employed to perform multiplexed Golden Gate Assembly and transform plasmids into *S. oneidensis* MR-1 through conjugation*. E. coli* WM3064 was cultured at 37 °C within LB broth, supplemented with 0.3 mM 2,6-Diaminopimelic acid (DAP). *S. oneidensis* MR-1 strains were cultivated aerobically at 30 °C in LB broth. Kanamycin (50 μg/mL) was added to LB broth as required for both *E. coli* and *S. oneidensis* MR-1. Isopropyl-β-d-thiogalactopyranoside (IPTG, 0.8 mM) was added in the medium as the inducer of P_tac_.

### 2.2 Plasmid construction

The plasmids used in this study are listed in Table S4. Sequences of Design Ⅰ-Ⅳ were synthesized and cloned into pCYR104 to generate pCYR293-296 by Genewiz (China). Sequence of P_J23119_-*rfp* flanked by two *Bsa*Ⅰ recognition sites (Table S5) were synthesized and cloned into pCYR104 to generate pMBE by Genewiz (China). Fourteen promoters with highly nonrepetitive sequence (Table S1) were synthesized and cloned into pCYR104 to generate pCYR277-289, 291 by Genewiz (China). Ten handles with highly nonrepetitive sequence (Table S2) were synthesized and cloned into pCYR104 to generate pCYR104-(h1-h10)-lacZ1 by Genewiz (China). Ten terminators with highly nonrepetitive sequence (Table S3) were synthesized and cloned into pCYR104 to generate pCYR104-lacZ1-(t1-t10) by Genewiz (China).

### 2.3 Real-time quantitative PCR (RT-qPCR)

Transcriptional level of a series of nonrepetitive promoters were detected. All strains were activated in 3 mL of LB medium with kanamycin for 12 h. Then, the culture suspension was inoculated into 50 mL of LB medium containing kanamycin and IPTG, incubating for 12 h. Total RNAs of cells were extracted using the Trizol reagent (Invitrogen, Carlsbad, CA, USA), according to the instructions of the manufacturer and treated with RQ1 DNase (Promega, Madison, WI, USA) to remove DNA. The quality and quantity of the purified RNA were determined by measuring the absorbance at 260 nm and 280 nm (A260 and A280) using a SmartSpec Plus Spectrophotometer (Bio-Rad Laboratories, Inc., Hercules, CA, USA). RNA integrity was further verified by electrophoresis using a 1.5% agarose gel. All RNA samples were stored at −80 °C for further use. Reverse transcription reactions were carried out using ReverTra Ace qPCR RT Kit (TOYOBO Life Science, Shanghai, China), according to the manufacturer's instructions.

The *gyrB* gene of *S. oneidensis* MR-1 was used as the endogenous reference gene for normalization. Specific primers were designed based on DNA sequences of sgRNA. Primer sequences used are listed in Table S6. The RT-qPCR was performed on a Bio-Rad S1000 with Bestar SYBR Green RT-PCR Master Mix (TOYOBO). PCR programme is consisted of denaturing at 95 °C for 1 min, and 40 cycles of denaturing at 95 °C for 15 s followed by annealing and extension at 60 °C for 30 s. Relative gene expression was calculated using the 2^−ΔΔCT^ method [35], normalized with the endogenous reference gene, *gyrB*. PCR amplifications were performed in triplicate for each sample.

### 2.4 One-pot assembly of multiplexed gRNAs

Firstly, the separate sgRNA cassettes were amplified by a series of primers (Table S6) via PCR with *Bsa*Ⅰ recognition sites and orthogonal overhangs that have been confirmed to enable Golden Gate assembly (Table S7). Secondly, Golden Gate rection in a PCR rection tube was performed as the former study with a little increased proportional weight of vector and fragment [36]. Thirdly, the rection setup was transformed into *E. coli* WM3064 and the transformants were spread on LB plates supplemented with DAP and kanamycin. Finally, one or two white clones were picked and then sequenced to confirm the insertion and correct sequences of sgRNAs.

### 2.5 Introduction of premature stop codon in lacZ

When *lacZ* was used as target gene, 60 μg/mL X-gal was added for blue-white selection. The plates were incubated at 30 °C for approximate 24 h until colonies appeared. The deactivation frequency of *lacZ* was calculated by counting colonies turning white. For each genotype (sgRNA with replaced promoter, handle or terminator), five strains that turned white (actually the original red color of *S. oneidensis* MR-1) were randomly selected, and the target locus was subsequently amplified and sequenced. The primers used for PCR amplification and sequencing are listed in Table S6. For convenience, one of the amplification primers was used for Sanger sequencing.

### 2.6 Multiplexed base editing in S. oneidensis MR-1

Plasmids harboring dCas9-AID and multiple gRNAs expression cassette were transformed into *S. oneidensis* MR-1 via conjugation with *E. coli* WM3064. The cells were recovered at 30 °C for 1–2 h and then spread on LB plates supplemented with kanamycin and IPTG. To testify the editing efficiency, the cells were purified by streaking and genome amplified and sequenced (n ≥ 8). Primers used for PCR amplification and sequencing are listed in Table S6. For convenience, one of the pair amplification primers was used for Sanger sequencing.

### 2.7 Construction of multiple engineered strains for enhancing RF- mediated EET

pMBE assembled with multiple gRNAs was removed after editing from *S. oneidensis* MR-1 as our previous report [21]. Then PYYDT-C5 (PYYDT-*ribADEHC*) plasmid was transformed into the edited strains to construct HRF(3BE) and HRF(8BE) via conjugation with *E. coli* WM3064. The cells were recovered at 30 °C for 1–2 h and then spread on LB plates supplemented with kanamycin and IPTG.

### 2.8 Bio-electrochemical characterization

Overnight HRF(3BE), HRF(8BE) and PYYDT-C5 strains culture suspensions were inoculated into 100 mL fresh LB broth supplemented with kanamycin and IPTG, 30 °C with shaking (200 rpm). After 10–12 h culture, the concentrations of cell suspensions were adjusted to the same level (OD_600_ = 0.5). All MFCs were incubated in 30 °C incubator, and each strain was tripled for parallel experiments. Dual-chamber MFCs with a working volume of 140 mL separated by the Nafion 117 membrane (DuPont Inc., United States) were harnessed in this work. Carbon cloth was used as the electrodes for both anode (1 cm × 1 cm, *i.e.*, the geometric area is 1 cm^2^) and cathode (2.5 cm × 3 cm). The anolyte is constituted by M9 buffer (Na_2_HPO_4_, 6 g/L; KH_2_PO_4_, 3 g/L; NaCl, 0.5 g/L; NH_4_Cl, 1 g/L; MgSO_4_, 1 mM; CaCl_2_, 0.1 mM), supplemented with 20 mM lactate and 5% (v/v) LB broth. The catholyte included 50 mM K_3_[Fe (CN)_6_], 50 mM KH_2_PO_4_ and 50 mM K_2_HPO_4_ solution. To measure the voltage of MFCs, a 2k external resistor was connected into the external electrical circuit, through which the output voltages were recorded automatically. Linear sweep voltammetry (LSV) analysis was performed with a scan rate (0.1 mV/s) as a two-electrode mode. The polarization curves were obtained by the counter electrodes for the assessment of the maximum power density. Power density (P) was calculated as P = V (output voltage) × I (current density). Both I and P were normalized to the projected area of the anode surface that is 1 cm^2^.

## 3 Results

### 3.1 Assessment of different strategies to express multiple gRNAs

Multiplexed CRISPR systems rely on the simultaneous and stable co-expression of multiple gRNAs [31], which can be implemented by expressing each gRNA under the control of an individual promoter or by producing a single transcript encoding multiple gRNAs, each separated by RNA cleavage sequences [25,31,37]. It has been generally accepted that the polycistronic approach provides a higher level of gRNA scalability and stoichiometry for CRISPR multiplexing compared to the monocistronic approach [22]. Nonetheless, polycistronic systems often require complex process to assembly sgRNAs or synthesis of multiple sgRNAs. In contrast, the monocistronic approach offers significant convenience in cloning methods because multiple sgRNAs can be directly integrated without additional gene flanking modifications. However, the monocistronic way contains compact repetitive DNA sequences in both the sgRNA structure, promoter and terminator [31,32]. Active homologous recombination may lead to gRNA loss through spontaneous deletion, triggering assembly difficulties and genetic instability [33,34]. Overall, both methods have their own advantages, with monocistrons being simpler to operate and polycistrons having the potential to edit more genes.

**Fig. 2:**
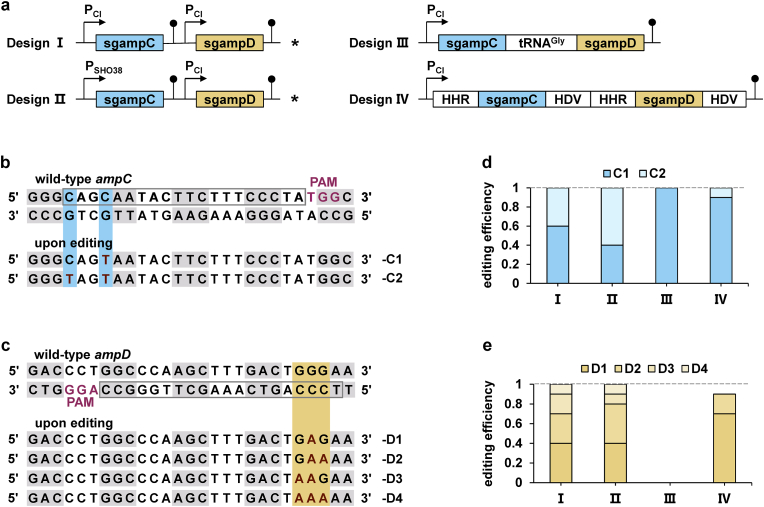
Assessment of different strategies to express multiple gRNAs. (a) Four designs to express gRNAs. Two genes, *ampC* and *ampD* were selected as the targets. HHR, Hammerhead ribozyme; HDV, hepatitis delta virus ribozyme. Asterisks represent the chosen designs for further investigation. The processing modes of Design Ⅲ and Ⅳ are shown in Figs. S1 and S2. (b) Mutated sequences obtained from editing event of *ampC* site. (c) Mutated sequences obtained from editing event of *ampD* site. (d) Editing efficiency of each mutated sequences of *ampC* site (n = 10). (e) Editing efficiency of each mutated sequences of *ampD* site (n = 10).

To test which method is more appropriate for base editing in *S. oneidensis*, we introduced 4 designs, including monocistronic (Design Ⅰ/Ⅱ) and polycistronic (Design Ⅲ/Ⅳ) (Fig. 2a). And the editing results of two sites were used as a reference to select the appropriate scheme. For monocistronic expression, the same promoter P_CI_ was adopted to control individual gRNA in Design Ⅰ, while two different promoters, P_SH038_ and P_CI_, were exploited in Design Ⅱ (Fig. 2a). For polycistronic expression, additional processing mechanisms are needed to generate functional gRNAs from a single array. In this regard, available options include flanking each gRNA with endogenous cleavage sequences tRNAs [26], self-cleavable ribozyme sequences [28,30],or by introducing exogenous RNA cleavage enzymes, such as Csy4, to process the primary RNA transcript [25]. Compared with endo- and self-cleavage, the introduction of exogenous cleavage proteins is obviously more complicated. Therefore, we pursed to exploit endo- and self-cleavage sequences for polycistronic expression of gRNAs. Processing of tRNAs is mediated by ribonucleases P and Z, cleaving near the 5′ and 3’ ends, respectively, of each tRNA [[38], [39], [40], [41]]. Since tRNA^Gly^ has been applied in gRNA processing in eukaryotes and prokaryotes and is relatively short with 71 base pairs (bp) compared to other endogenous tRNAs [26,[42], [43], [44]], Design Ⅲ uses tRNA^Gly^ (endogenous in *S. oneidensis*) to link two sgRNAs, which is a simple and compact architecture (Fig. 2a and S1). Likewise, Design Ⅳ uses popular self-cleavable ribozyme sequences (Hammerhead ribozyme (HHR) and hepatitis delta virus (HDV) ribozyme) to process the transcripts (Fig. 2a and S2). Given that HHR and HDV each can only cleave one fixed site, to fully release sgRNA, each sgRNA needs to be flanked by two ribozyme sequences, which makes the genetic structure of Design Ⅳ more complex than Design Ⅲ.

Our previously developed CRISPR/dCas9-AID was employed as the base editor, due to its high efficiency and low toxicity in *S. oneidensis* [21]. Meanwhile, to avoid editing efficiency bias of different gRNA, two gRNAs, sgampC and sgampD were selected from our previous work [21], which both showed 100% editing efficiency for single site editing (Fig. 2a). Ten strains were randomly selected for sequencing of each design to verify multiplex editing efficiency. The results of editing *ampC* and *ampD* are shown in Fig. 2b and **c**. Editing efficiency of both loci for Design Ⅰ and Ⅱ was 100% (n = 10). The efficiency of editing *ampC* for Design Ⅳ was 100%, and efficiency of editing *ampD* was 90% (n = 10) (Fig. 2d and **e**). While Design Ⅲ failed to edit *ampD*, which is targeted by the second sgRNA, indicating that sgRNA array separated by tRNA^Gly^ was inappropriate for multiplexed base editing in *S. oneidensis* (Fig. 2e). Overall, expressing each sgRNA as a monocistronic unit is more favorable for multiplexed base editing in *S. oneidensis*, either using the same promoter or different promoters (Fig. 2d and e).

### 3.2 Highly efficient multiplexed base editing

**Fig. 3:**
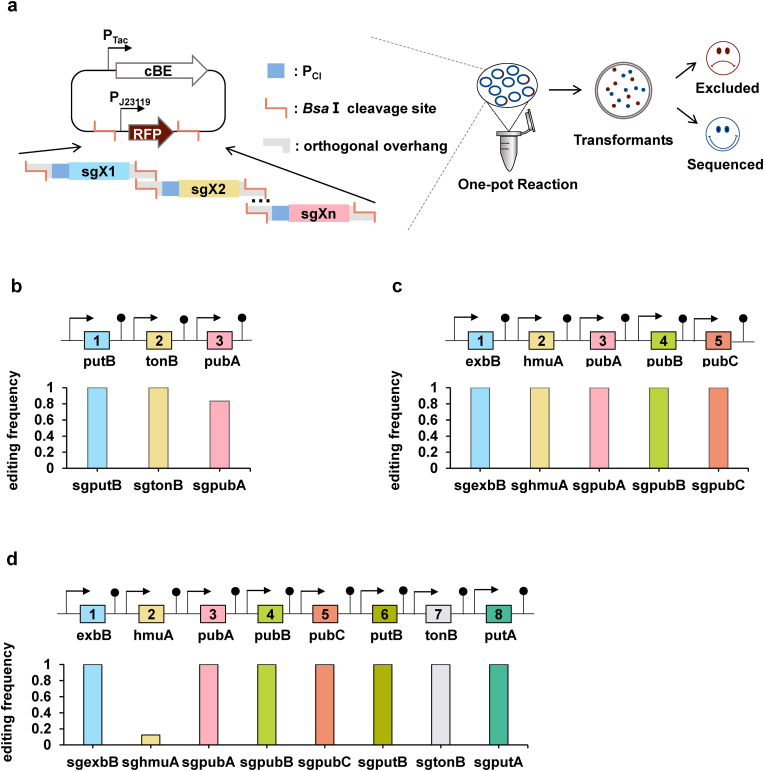
Rapid and smart multiplexed base editing with high efficiency. (a) One-step assembly strategy to access the multiplexed base editing plasmid. RFP was employed as the reporter to confirm the insertion of gRNAs. The *E. coli* strain in red can be excluded as the negative clones. (b) Editing efficiency of targeting 3 endogenous genes (n = 12). (c) Editing efficiency of targeting 5 endogenous genes (n = 12). (d) Editing efficiency of targeting 8 endogenous genes (n = 8). The corresponding editing sequence results of (b) and (d) are shown in Figs. S5 and S6.

To obtain single DNA construct for multiplexing gRNAs, three ways haven been applied already, stepwise assembly [37], one-pot assembly [45], and direct synthesis [24]. The low throughput procedures of stepwise assembly are visibly demanding when it comes to more than 3 genes [21]. Array-based gene synthesis is viable, due to its facile operation [24,46]. Nevertheless, inflexibility makes it inaccessible in the practical application when different combinations need to be tested. One-pot assembly strategy is inexpensive and highly malleable for carrying out multiple base editing in *S. oneidensis*. We have proved that monocistronic expression of each sgRNA is suitable for expression of multiple gRNAs in *S. oneidensis*, either controlled by the same promoter or the different. In this section, the same promoter P_CI_ would be exerted firstly [21]. And a smart one-step assembly strategy was designed to access the multiplexed base editing plasmid. As shown in Fig. 3a, multiple gRNAs was cloned into vector pMBE by Golden Gate method [47], supported by employing a *rfp* as the reporter gene under control of constructive promoter P_J23119_ to confirm the insertion. In the process of ligating multiple gRNAs, the *E. coli* strain in red can be excluded as the negative clone, leading to a marked increase in the proportion of positive clones (Fig. 3a).

The editing efficiency was testified via deactivation of 3, 5, and 8 endogenous genes by our system (Fig. 3b–d). Strains harboring pMBE inserted with multiple gRNAs were induced by IPTG for 12h and purified as the former study [21]. The editing region was amplified and Sanger sequenced for each target. For the deactivation of 3 genes, of the 12 strains that were amplified and sequenced, 10 strains successfully edited all 3 genes, resulting in an editing efficiency of 83%, while the other 2 strains edited only 2 genes. For the deactivation of 5 genes, all 12 strains were successfully edited with 100% editing efficiency. These results indicated that our system enabled multiplexed base editing with dramatically high efficiency (Fig. 3b and c). For the deactivation of 8 genes, one of the 8 sequenced strains was successfully edited, while the other 7 strains all had one locus editing failure (Fig. 3d). To our best knowledge, this is the first successful attempt to generate deactivation of 8 genes at once in *S. oneidensis* MR-1.

Overall, the multiplexed base editing system we developed here is able to obtain multigene deactivated strains rapidly and efficiently. In fact, strategies of expression and assembly of multiple gRNAs can be compatibly transferred to other CRISPR-based technologies, such as CRISPR activation and interference [10], which would facilitate advances in genetic tools in terms of synthetic biology modification throughput in *S. oneidensis*.

### 3.3 Screening of nonrepetitive genetic parts of sgRNA cassette

**Fig. 4:**
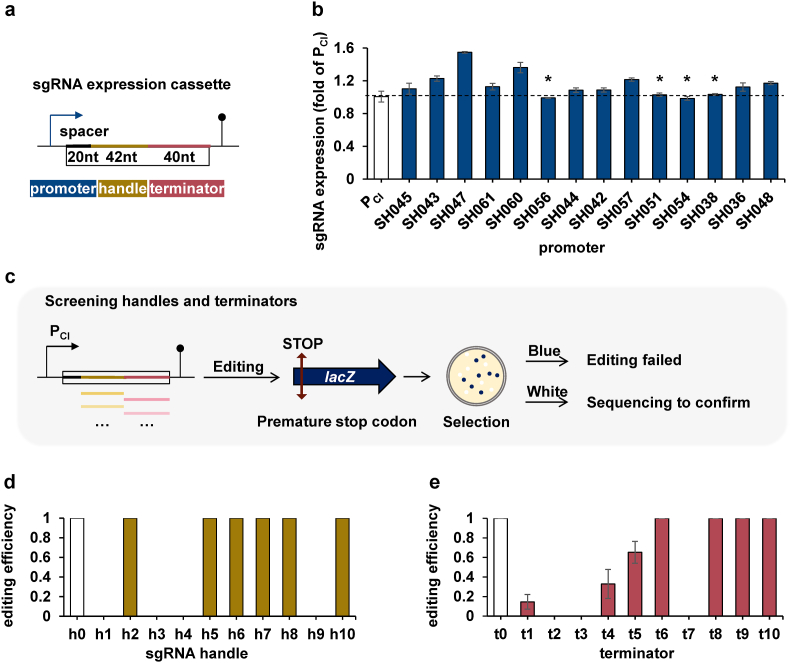
Screening of nonrepetitive genetic parts of sgRNA cassette. (a) Schematic representation of the nonrepetitive components to be screened of sgRNA cassette, including promoter, handle and terminator. (b) Transcriptional level of sglacZ1 under control of the promoter candidates. Asterisks represent the promoters that showed the closest expression level to P_CI_. The editing efficiency of sglacZ1 controlled by P_SH056_, P_SH054_, P_SH051_, and P_SH038_ were shown in Fig. S3. (c) Screening of nonrepetitive handles and terminators using *lacZ* as the target for introduction of premature stop codon. (d) Editing efficiency of CRISPR/dCas9-P_CI_-sglacZ1 with replaced handles. (e) Editing efficiency of CRISPR/dCas9-P_CI_-sglacZ1 with replaced terminators. Values and error bars indicate mean ± standard error of mean (s.e.m.) of three replicates.

Although our multiplexed base editor already enabled efficient and high-throughput gene deactivation by using the same promoter to express multiple gRNAs, this system contains several DNA repeats in promoter, terminator and sgRNA structure [31,32]. In fact, we found two practical difficulties in our operation: i) relatively low cloning success rate; ii) low sequencing accuracy. Both of these problems are caused by the presence of repetitive sequences that induce homologous recombination. It has been demonstrated that CRISPR multiplexity can be advanced by designing toolboxes with highly nonrepetitive genetic parts. We therefore selected a serial of nonrepetitive genetic units of sgRNA expression cassette, including promoters, handles, and terminators from a seminal work [24], and tested the utility of these components for base editing in *S. oneidensis* (Fig. 4a).

Fourteen promoters that do not share more than 22 base pairs (bp) of identical contiguous DNA sequence were chosen to control the expression of sgRNA (Table S1). In order to edit multiple targets unbiasedly, equimolar gRNA expression and thus the promoters with similar expression strengths are needed [48]. Our previous work has proven that sgRNA under control of promoter P_CI_ enables highly efficient base editing of CRISPR/dCas9-AID system in *S. oneidensis* [21]. Accordingly, promoters that can obtain similar expression to P_CI_ are suitable candidates for the construction of nonrepetitive gRNAs array. To compare the expression strength of candidate promoters with that of P_CI_, a previously proven effective sgRNA, sglacZ1 [21], was cloned downstream of each promoter, and their expression levels were then measured by RT-qPCR. As shown in Fig. 4b, the sgRNA expression levels of the candidate promoters were 0.98–1.5-fold compared to P_CI_, indicating that the intensity difference between these promoters was not significant. The sgRNA expression of P_SH056_, P_SH054_, P_SH051_, and P_SH038_ were 0.99-, −0.98-, 1.03-, 1.03-fold of that of P_CI_, respectively, which were the closest in expression level to P_CI_ (Fig. 4b). Then the editing outcomes of the base editor with sgRNA controlled by these four promoters were confirmed, as our expected, all the editing efficiency were 100% (n = 10) (Fig. S3). Therefore, they should preferentially be selected for the construction of nonrepetitive gRNAs array. In addition to express sgRNA, these promoters have enriched synthetic biology genetic devices of *S. oneidensis* for EET studies.

For sgRNA handle, ten candidates that avoid sharing more than 20 bp of the same consecutive DNA sequence were selected to substitute the classical 42-bp handle (Table S2). To easily evaluate the effectiveness of the sgRNA harboring these handles for base editing in *S. oneidensis*, sgRNA cassettes of P_CI_-sglacZ1 with replaced handles were constructed, and *lacZ* in genome (JG2150) [13] was used as the target for introduction of premature stop codon (Fig. 4c). Cells harboring dCas9-AID and sgRNA with different handle were coated on X-gal and IPTG supplemented plates. Six of ten handles (h2, h5-8, h10) showed 100% editing frequency (n = 3) (Fig. 4d). For each handle-changed sgRNA, five strains that turned white (actually the original red color of *S. oneidensis* MR-1) were randomly selected, and the target locus was subsequently amplified and sequenced. The results showed that all the turning-white strains were edited successfully, suggesting that these 6 handles are adapted to base editing in *S. oneidensis*.

Meanwhile, ten candidates of terminators that share no more than 12 bp of the same consecutive DNA sequence were selected to replace the original *S. pyogenes* terminator (Table S3). Similar to the screening of handle alternatives, sgRNA cassettes of P_CI_-sglacZ1 with substituted terminators were constructed, and *lacZ* in genome [13] was used as target (Fig. 4c). The editing results of blue-white selection showed that four terminators of t6, t8, t9 and t10 had an editing frequency of 100% (n = 3), which should be chosen in preference (Fig. 4e). There are also sgRNAs employing other terminators that can succeed in mediating base editing, indicating that these terminators can be used as alternatives when needed (Fig. 4e).

Taken together, we proposed a series of nonrepetitive alternatives for each genetic part of original sgRNA sequence. These alternatives make it possible to avoid the difficulties of assembly and sequencing as well as the genetic instability caused by repetitive sequences of multiple sgRNAs. The same and the different promoters could be employed in a blend manner in future, offering opportunity for further augmenting the expression of multiplexed gRNAs and to construct chassis strains with more deactivated genes.

### 3.4 Deactivation of 8 genes to enhance riboflavin-mediated EET via multiplexed base editing

Efforts of metabolic engineering and synthetic biology allow the rational modulation of the numerous factors affecting the EET efficiency, including intracellular redox conditions, electron transfer shuttle biosynthesis, conductive pili construction, and biofilm formation and maturation [7,49]. Apart from the processes directly related to EET, seemingly irrelevant cellular activities and physiological features also have an effect on the efficiency of EET, which is constantly overlooked. The time-consuming and labor-intensive genetic modification further hinders the study of the association of these genes with EET [50]. Albeit base editing has considerably expedited the genome manipulation of *S. oneidensis* MR-1, multiple iterations of editing still preclude the evolution of complex mechanism elucidation, regulation pathway identification and EET potential mining. By virtue of multiplexed base editing system, the capacity of silencing more genes simultaneously would be conferred to *S. oneidensis* MR-1, thereby helping to elevate our understanding of EET.

*S. oneidensis* exchanges electron with electron acceptors through two modes, direct electron transfer (DET) and mediated electron transfer (MET) [51]. DET is mainly relied on cytochromes, and MET uses electron transfer shuttles. Riboflavin (RF) plays a central role in the EET process of *S. oneidensis*, embodied in the following three aspects: (i) acting as the electron shuttle, largely determining the rate of MET [52]; (ii) combining the outer-membrane *c*-type cytochromes as redox cofactor to enhance the efficiency of DET [53,54]; (iii) triggering anaerobic biofilm formation, which is the paramount basis of both MET and DET [55]. To demonstrate the feasibility of multiplexed base editing system for combinatorial engineering applications of EAMs, our sights were set onto RF-mediated EET.

**Fig. 5:**
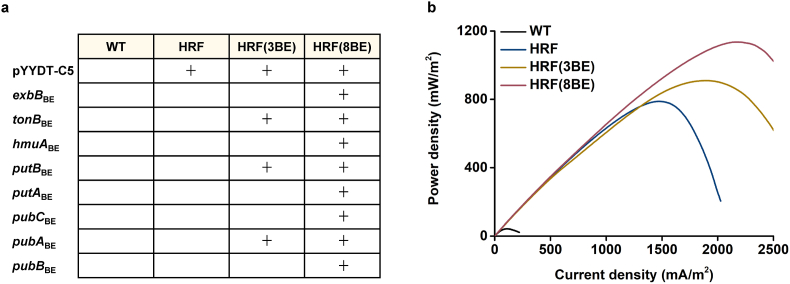
Deactivation of 8 genes to enhance riboflavin-mediated EET via multiplexed base editing. (a) Schematic illustration of multiplex engineered strains with deactivated genes. “+” stands for deactivating of corresponding gene or harboring corresponding plasmid. WT represents the wild type *S. oneidensis* MR-1. PYYDT-C5 represents PYYDT plasmid with *ribADEHC*, a gene cluster encoding riboflavin biosynthetic pathway originated from *Bacillus subtilis*. The riboflavin biosynthesis pathway was shown in Fig. S4. HRF, high-production riboflavin. (b) Power density output curves obtained by linear sweep voltammetry (LSV) with a scan rate of 0.1 mV/s. The maximum power densities of multiplex engineered strains were shown in Fig. S7.

Considerable efforts have been made to modulate the flavin biosynthetic pathway in order to heighten RF-mediated EET [56,57]. Recently, a number of genes indirectly associated with RF-mediated EET have been identified by comparative transcriptome analyses, which were conducted between a RF-overproducing strain and a strain carrying empty plasmid [58]. Among the significantly downregulated genes candidates, the effectiveness of silence of each gene has been determined by the bio-electrochemical analysis [58]. As a next step, we here leveraged multiplexed base editing for combined reverse engineering, thus these genes were deactivated in combination for reinforcing RF-mediated EET. According to the results of comparative transcriptome analyses, the most significantly downregulated genes, 8 in total, were selected as our targets (Fig. 5a). As the candidate genes were identified in the context of high-RF production, the gene cluster *ribADEHC* (derived from *Bacillus subtilis*) encoding RF biosynthetic pathway was overexpressed to confer high RF generation capacity in *S. oneidensis* MR-1 (Fig. S4). This strain was termed as HRF, and above this strain, HRF(3BE) (*tonB*_BE_-*putB*_BE_-*pubA*_BE_- PYYDT-C5) and HRF(8BE) (*tonB*_BE_-*putB*_BE_-*pubA*_BE_-*exbB*_BE_-*hmuA*_BE_-*putA*_BE_-*pubC*_BE_-*pubB*_BE-_ PYYDT-C5) were constructed (Figs. S5 and S6). To verify the EET outputs of the multiplex engineered strains, HRF(3BE), HRF(8BE), PYYDT-C5 and WT were cultured in MFCs. The bio-electrochemical analysis was then conducted during the plateau of voltage (Fig. 5b). The power density curves showed that the maximum power density of HRF(3BE) and HRF(8BE) reached 909.47 ± 196.79 mW/m^2^ (value ± SD, n = 3) and 1108.1 ± 117.5 mW/m^2^ (value ± SD, n = 3), which were 17.79 and 21.67 folds higher than that of the WT strain (51.12 ± 14.90 mW/m^2^, value ± SD, n = 3), respectively (Fig. 5b and S7). Meanwhile, the maximum power density of HRF(3BE) and HRF(8BE) were 1.24 and 1.51 folds higher than the PYYDT-C5 strain (731.53 mW/m^2^ ± 130.474 mW/m^2^, value ± SD, n = 3) (Fig. 5b and S7). The results indicated that the electron transfer rate of the high-level chassis could also be substantially improved by deactivating multiple genes via the base editing system.

Our previous work showed that the individual deactivation of gene *exbB*, *hmuA*, *putA*, *pubC*, and *pubB* had no significant helpful effect on the enhancement of EET [58]. Nonetheless, the maximum power density of HRF(8BE) was ∼1.22 folds higher than that of HRF(3BE), suggesting that combinatorial modification of genes that do not have positive effects alone might have unexpected outcome. This example of multiple genomic manipulation corroborated that multiplexed base editing empowered *S. oneidensis* with robust gene editing capability, offering huge potential to study the relation between multiple genes and the efficiency of EET.

## 4 Discussion

Base editing has been fully exploited not only within model microorganisms but also within many industrially, agriculturally, and clinically important non-model microorganisms due to its simple and programmable properties that do not require the generation of DNA double-strand breaks [59]. In fact, many of the multiplex base editing tools of non-model organisms have been implemented in a simple and straightforward manner-monocistronic approach to express gRNAs [[60], [61], [62], [63], [64], [65], [66]]. There are certainly some outstanding works that use polycistronic approach to express gRNAs, such as the recently reported multiple gene editing achieved in *Pseudomonas* spp, which can simultaneously edit 9 targets [67]. Since an exogenous cleavage enzyme is used to process the gRNAs, the system requires an additional nucleic acid endonuclease (Csy4), but still multiple editing was achieved with only one plasmid. And it can be exploited not only in *P*. *putida*, but also in *P*. *aeruginosa* and *E. coli*. Since a broad-host plasmid is employed, it seems likely that this base editor can be applied in other Gram-negative bacteria as well. Although our system uses broad-host replicon PBBR1, it remains to be verified whether it can function in other species.

Differences in multiplexed editing efficiency may stem from whether gRNAs array are processed into clean and accurate sgRNAs for polycistronic systems. The self-cleavable ribozyme sequences can theoretically produce precise cleavage in any species, whereas we were unsure that the cleavage site of the endogenous cleavage enzyme recruited by tRNA^Gly^ was identical to the cleavage site of the model bacterium, *E. coli*. Based on the editing performance of Design III (Fig. 2d and e), we speculated that the reason for the failure of Design III to edit *ampD* could be the inaccurate cleavage of the 3′ end of the tRNA^Gly^, resulting in redundant sequences at the 5′ end of the released second sgRNA (sgampD), which is a fatal problem for any type of CRISPR-mediated system. We also observed that not all possible edits occurred in strains that were randomly picked for sequencing (Fig. 2b and c). The results indicated that each locus has an editing preference and also implied that those editing results that did not occur were not high-frequency events. The absence of some possible outcomes is determined by the sample size. If the sample size is large enough, theoretically any possible editing result can be detected, especially when next-generation sequencing (NGS) is used.

Interestingly, Design Ⅰ and Ⅱ yielded more diversified editing results, with more Cs being mutated into Ts compared to Design Ⅳ (Fig. 2d and e). The variable of the different designs (Ⅰ, Ⅱ and Ⅳ) is the amount of sgRNA. We thus speculated that strains harboring monocistronic systems contained more sgRNAs than strains harboring polycistronic system, which directly increased the probability that dCas9-AID bound to the target genes, resulting in more edited Cs. Considering that Design I and IV use the same promoter, there may be some gRNAs array to be processed in Design IV, resulting in fewer sgRNAs available than Design I and II.

We selected monocistronic approach to express gRNAs through testing editing efficiency of two sgRNAs, which does not mean that polycistronic approach cannot be applied to the multiplexed CRISPR system in *S. oneidensis*. Conversely, we believe that when the number of gRNAs is larger than 8, the polycistronic method may show advantages over monocistronic method due to its scalability. Meanwhile, monocistronic approach may also face issues with promoter crosstalk. Having successfully achieved multiple editing employing the same promoter, we would like to provide some more nonrepetitive genetic parts for use when more than 8 genes need to be edited or when gRNAs are really difficult to assemble.

Because of the safety concerns of CRISPR application in mammalian cells, off-target effects have received extensive attention [68]. In this regard, genome editing of microorganisms as well should avoid off-target effects as much as possible, especially for strains that need to be released into the environment to function. Due to the presence of effector cytidine deaminase, base editing carries not only the off-target risk of CRISPR system, but also cytidine deaminase-induced off-target editing, which has been reported in multiple species [[69], [70], [71]]. Therefore, in environmental applications and mechanism studies, we recommend selecting sgRNAs with low off-target probability through sgRNA design websites (such as http://crispor.org) [72], and performing whole-genome sequencing to confirm editing specificity before application.

## 5 Conclusions

Herein, a rapid and highly-efficient multiplexed base editing system was constructed in the EAM model strain *S. oneidensis* MR-1. We evaluated the methods to express multiple gRNAs and designed a smart one-pot scheme to assemble gRNAs. Efficient simultaneous editing of 3, 5 and 8 genes were achieved. In addition, to address the gene instability caused by repetitive sequences in the sgRNA cassette, we screened a series of highly nonrepetitive components, including promoters, handles and terminators. Eight genes that were identified as targets of significant down-regulation in transcriptome analysis of riboflavin overproducing strain and control strain were simultaneously deactivated using this multiplexed base editor. The engineered strain showed considerably improved EET efficiency, compared to the parental strain. Our results demonstrate that a single editing event enables the acquisition of 8x gene deactivations, which represents a powerful addition to the synthetic biology toolkit for EAMs. Regarding to the gene-function connection and gene-gene connection, researchers understand *S. oneidensis* far less well than other model microorganisms [1]. Development of genome-scale perturbation, complementary various omics, and high-throughput sequencing technology has enabled a lot of gene targets discovered [73]. In this case, multiplexed base editing system offers an opportunity to simultaneously investigate numerous genes, which would facilitate both applied and basic EET research.

## CRediT authorship contribution statement

**Yaru Chen:** Conceptualization, Methodology, Formal analysis, Investigation, Writing – original draft, Writing – review & editing, Visualization. **Meijie Cheng:** Investigation, Formal analysis. **Yan Li:** Investigation, Formal analysis, Visualization. **Lin Wang:** Investigation. **Lixia Fang:** Methodology, Writing – review & editing. **Yingxiu Cao:** Writing – original draft, Conceptualization, Supervision, Funding acquisition. **Hao Song:** Writing – original draft, Conceptualization, Supervision, Funding acquisition.

## Declaration of competing interest

The authors declare that they have no known competing financial interests or personal relationships that could have appeared to influence the work reported in this paper.
