# Supplementary material for: Highly efficient multiplex base editing: One-shot deactivation of eight genes in Shewanella oneidensis MR-1
Source: Synth Syst Biotechnol. 2022 Oct 13;8(1):1–10. doi: 10.1016/j.synbio.2022.09.005 (PMC9594123; doi:10.1016/j.synbio.2022.09.005)
Supplement: Multimedia component 1 [file mmc1.docx]

Supplementary Information

**Highly efficient multiplex base editing: one-shot deactivation of eight genes in *Shewanella oneidensis* MR-1**

Yaru Chen^1,2^, Meijie Cheng^1,2^, Yan Li^1,2^, Lin Wang^1,2^, Lixia Fang^1,2^, Yingxiu Cao^1,2,^* and Hao Song^1,2,^*

^1^ Frontier Science Center for Synthetic Biology and Key Laboratory of Systems Bioengineering (Ministry of Education), School of Chemical Engineering and Technology, Tianjin University, Tianjin 300072, China

^2^ Key Laboratory of Systems Bioengineering (Ministry of Education), Tianjin University, Tianjin 300072, China

*Correspondence: caoyingxiu@tju.edu.cn (Y.X.C.), hsong@tju.edu.cn (H.S.)

**Table S1.** Sequences of nonrepetitive promoters used in this study^1^

| ID | Sequence |
| --- | --- |
| P_CI_ | TAACACCGTGCGTGTTGACTATTTTACCTCTGGCGGTGATAATGGTTGC |
| P_SH045_^1^ | TTGACAACTGCTCAGCGAAATACTATAATGACTAC |
| P_SH043_^1^ | TTGACACTACCGAGACAGTGACATATAATAGGACC |
| P_SH047_^1^ | TTGACACTGGCCTGACAAGTCCATATAATGATGTC |
| P_SH061_^1^ | TTGACAGAGGCAGTACTACCGTTTATAATTCGGAC |
| P_SH060_^1^ | TTGACATTAGCACTTGAGCTGATTATAATGGGCCG |
| P_SH056_^1^ | TTGACACTTTGCACATGTCCCGTTATAATCATGAT |
| P_SH044_^1^ | TTGACACGATGCTTGCTGCTACCTATAATAACATA |
| P_SH042_^1^ | TTGACAGTAGATCAGAGGGTTGCTATAATCGACAG |
| P_SH057_^1^ | TTGACACGGATCTTCGCTGAACGTATAATGAGAAA |
| P_SH051_^1^ | TTGACATAAGTCGTATTCAAAGATATAATATAGGT |
| P_SH054_^1^ | TTGACAGCATCTGCTTTGTCACCTATAATTCAATG |
| P_SH038_^1^ | TTGACATGAGCTCGTCGTCAGGATATATAGCTTT |
| P_SH036_^1^ | TTGACATGACTCTCCAGCTGTGCTATAATTGTACT |
| P_SH048_^1^ | TTGACACTATGGTCCGCAAGCATTATAATGCTCTG |

**Table S2.** Sequences of nonrepetitive handles of gRNA used in this study

| ID | Sequence |
| --- | --- |
| h0 | GTTTTAGAGCTAGAAATAGCAAGTTAAAATAAGGCTAGTCCG |
| h1^1^ | GTTCTAGAGCTGGTAACAGCAAGTTAGAATAAGTCTAGTCCA |
| h2^1^ | GTTTTAGAGTGAGAAATCACAAGTTAAAATAAGGCTAGACCG |
| h3^1^ | GTTGTAGAGCTAGCAATAGCAGGTTACAATAAGGCTCGTCCG |
| h4^1^ | GATTTCGAGCTAGGCATAGCAAGTGAAATTAAGGCTGGTCCA |
| h5^1^ | GATTTAGAGCTGGAAACAGCAAGTTAAATTAAGGCTAGTCCG |
| h6^1^ | GTTGTAGAGGAAGAAATTCCAAGTTACAATGAGGCTAGTCCG |
| h7^1^ | TTTTCAGATTTGGAAACAAAACGTTGAAAAAAGGCAAGTCCG |
| h8^1^ | GATGTAGATGTAGAAATACAAGGTTACATTAAGGCCCGTCCG |
| h9^1^ | GTTTTGGACCTAGAAATAGGAAGTCAAAATAAGGCTGGACCG |
| h10^1^ | GTTGGAGAGCAAGACATTGCAAGTTCCAATAAGGCGTGTCCG |

**Table S3.** Sequences of nonrepetitive terminators used in this study

| ID | Sequence |
| --- | --- |
| t0 | TTATCAACTTGAAAAAGTGGCACCGAGTCGGTGCTTTTTTT |
| t1^1^ | AGAAAAAAGCCCGCACCTGACAGTGCGGGCTTTTTTTTTCGA |
| t2^1^ | ATGAGAAAGCCCCCGGAAGATCACCTTCCGGGGGCTTTTTTATTGCG |
| t3^1^ | AAAAAAAATCCTTAGCTTTCGCTAAGGATGATTTCTACTA |
| t4^1^ | AGTCAAAAGCCTCCGGTCGGAGGCTTTTGACTTTCT |
| t5^1^ | TGAAAAAGCCCGCGCAAGCGGGTTTTTTTATGAC |
| t6^1^ | GCACAAAAGCCAGTCTGGAAACAGGCTGGCTTTTTTTTGCGA |
| t7^1^ | CATTCTTAGCGTGACCGGGAAGTCGGTCACGCTACCTCTTCTGA |
| t8^1^ | TTCCATCGGGTCCGAATTTTCGGACCTTTTCTCCGCAC |
| t9^1^ | AAAAGAGACGCTTTTAGAGCGTCTTTTTTCGTT |
| t10^1^ | AACAATAAGGGGAGCGGGAAACCGCTCCCCTTTTTTATTGAT |

**Table S4.** Strains and plasmids used in this study

| Strain or plasmid | Description^a)^ | Reference or source |
| --- | --- | --- |
| Strain | | |
| *E. coli* DH5α | General cloning host | Lab stock |
| *E. coli* Trans1-T1 | General cloning host | Lab stock |
| *E. coli* WM3064 | A DAP auxotroph of *E. coli* could transfer plasmid into *S. oneidensis* MR-1 by conjugation | Lab stock |
| *S. oneidensis* MR-1 | Wild-type strain | Lab stock |
| JG2150 | MR-1 derivative with insertion of a *lacZ* cassette | ^2, 3^ |
| HRF | MR-1 derivative with plasmid PYYDT-C5 | ^4^ |
| HRF(3BE) | MR-1 derivative with premature stop in gene *tonB*(Q197*), *putB*(Q45*), *pubA*(Q117*), and transformed with plasmid PYYDT-C5 | [This study](file:///C:\Users\dai'er\Desktop\TableS2.xlsx#RANGE!_ENREF_1) |
| HRF(8BE) | MR-1 derivative with premature stop in gene *tonB*(Q197*), *putB*(Q45*), *pubA*(Q117*), *exbB*(Q90*), *hmuA*(Q35*), *putA*(Q391*), *pubC*(Q40*), *pubB*(Q42*), and transformed with plasmid PYYDT-C5 | [This study](file:///C:\Users\dai'er\Desktop\TableS2.xlsx#RANGE!_ENREF_1) |
| Plasmid | | |
| pYYDT-C5 | *reppBBR1*, Kan^R^, *mob*, P_tac_-*ribADEHC* | ^4^ |
| pCYR104 | *reppBBR1*, Kan^R^, *oriT*, P_tac_-dCas9-AID, P_CI_-sgRNA | ^5^ |
| pCYR293 | pCYR104 derivative, Design Ⅰ, P_CI_-sgampC, P_CI_-sgampD | [This study](file:///C:\Users\dai'er\Desktop\TableS2.xlsx#RANGE!_ENREF_1) |
| pCYR294 | pCYR104 derivative, Design Ⅱ, P_SH038_-sgampC, P_CI_-sgampD | [This study](file:///C:\Users\dai'er\Desktop\TableS2.xlsx#RANGE!_ENREF_1) |
| pCYR295 | pCYR104 derivative, Design Ⅲ, P_CI_-sgampC-tRNA^Gly^-sgampD | [This study](file:///C:\Users\dai'er\Desktop\TableS2.xlsx#RANGE!_ENREF_1) |
| pCYR296 | pCYR104 derivative, Design Ⅳ, P_CI_-HHR-sgampC-HDV-HHR-sgampD-HDV | [This study](file:///C:\Users\dai'er\Desktop\TableS2.xlsx#RANGE!_ENREF_1) |
| pMBE^b)^ | pCYR104 derivative, P_CI_-sgRNA::P_J23119_-*rfp* | [This study](file:///C:\Users\dai'er\Desktop\TableS2.xlsx#RANGE!_ENREF_1) |
| pCYR277 | pCYR104 derivative, P_CI_-sgRNA::P_SH045_-sglacZ1 | [This study](file:///C:\Users\dai'er\Desktop\TableS2.xlsx#RANGE!_ENREF_1) |
| pCYR278 | pCYR104 derivative, P_CI_-sgRNA::P_SH043_-sglacZ1 | [This study](file:///C:\Users\dai'er\Desktop\TableS2.xlsx#RANGE!_ENREF_1) |
| pCYR279 | pCYR104 derivative, P_CI_-sgRNA::P_SH047_-sglacZ1 | [This study](file:///C:\Users\dai'er\Desktop\TableS2.xlsx#RANGE!_ENREF_1) |
| pCYR280 | pCYR104 derivative, P_CI_-sgRNA::P_SH061_-sglacZ1 | [This study](file:///C:\Users\dai'er\Desktop\TableS2.xlsx#RANGE!_ENREF_1) |
| pCYR281 | pCYR104 derivative, P_CI_-sgRNA::P_SH060_-sglacZ1 | [This study](file:///C:\Users\dai'er\Desktop\TableS2.xlsx#RANGE!_ENREF_1) |
| pCYR282 | pCYR104 derivative, P_CI_-sgRNA::P_SH056_-sglacZ1 | [This study](file:///C:\Users\dai'er\Desktop\TableS2.xlsx#RANGE!_ENREF_1) |
| pCYR283 | pCYR104 derivative, P_CI_-sgRNA::P_SH044_-sglacZ1 | [This study](file:///C:\Users\dai'er\Desktop\TableS2.xlsx#RANGE!_ENREF_1) |
| pCYR284 | pCYR104 derivative, P_CI_-sgRNA::P_SH042_-sglacZ1 | [This study](file:///C:\Users\dai'er\Desktop\TableS2.xlsx#RANGE!_ENREF_1) |
| pCYR285 | pCYR104 derivative, P_CI_-sgRNA::P_SH057_-sglacZ1 | [This study](file:///C:\Users\dai'er\Desktop\TableS2.xlsx#RANGE!_ENREF_1) |
| pCYR286 | pCYR104 derivative, P_CI_-sgRNA::P_SH051_-sglacZ1 | [This study](file:///C:\Users\dai'er\Desktop\TableS2.xlsx#RANGE!_ENREF_1) |
| pCYR287 | pCYR104 derivative, P_CI_-sgRNA::P_SH054_-sglacZ1 | [This study](file:///C:\Users\dai'er\Desktop\TableS2.xlsx#RANGE!_ENREF_1) |
| pCYR288 | pCYR104 derivative, P_CI_-sgRNA::P_SH038_-sglacZ1 | [This study](file:///C:\Users\dai'er\Desktop\TableS2.xlsx#RANGE!_ENREF_1) |
| pCYR289 | pCYR104 derivative, P_CI_-sgRNA::P_SH036_-sglacZ1 | [This study](file:///C:\Users\dai'er\Desktop\TableS2.xlsx#RANGE!_ENREF_1) |
| pCYR291 | pCYR104 derivative, P_CI_-sgRNA::P_SH048_-sglacZ1 | [This study](file:///C:\Users\dai'er\Desktop\TableS2.xlsx#RANGE!_ENREF_1) |
| pCYR104-h1-lacZ1 | pCYR104 derivative, P_CI_-sglacZ1 with replaced handle h1 | [This study](file:///C:\Users\dai'er\Desktop\TableS2.xlsx#RANGE!_ENREF_1) |
| pCYR104-h2-lacZ1 | pCYR104 derivative, P_CI_-sglacZ1 with replaced handle h2 | [This study](file:///C:\Users\dai'er\Desktop\TableS2.xlsx#RANGE!_ENREF_1) |
| pCYR104-h3-lacZ1 | pCYR104 derivative, P_CI_-sglacZ1 with replaced handle h3 | [This study](file:///C:\Users\dai'er\Desktop\TableS2.xlsx#RANGE!_ENREF_1) |
| pCYR104-h4-lacZ1 | pCYR104 derivative, P_CI_-sglacZ1 with replaced handle h4 | [This study](file:///C:\Users\dai'er\Desktop\TableS2.xlsx#RANGE!_ENREF_1) |
| pCYR104-h5-lacZ1 | pCYR104 derivative, P_CI_-sglacZ1 with replaced handle h5 | [This study](file:///C:\Users\dai'er\Desktop\TableS2.xlsx#RANGE!_ENREF_1) |
| pCYR104-h6-lacZ1 | pCYR104 derivative, P_CI_-sglacZ1 with replaced handle h6 | [This study](file:///C:\Users\dai'er\Desktop\TableS2.xlsx#RANGE!_ENREF_1) |
| pCYR104-h7-lacZ1 | pCYR104 derivative, P_CI_-sglacZ1 with replaced handle h7 | [This study](file:///C:\Users\dai'er\Desktop\TableS2.xlsx#RANGE!_ENREF_1) |
| pCYR104-h8-lacZ1 | pCYR104 derivative, P_CI_-sglacZ1 with replaced handle h8 | [This study](file:///C:\Users\dai'er\Desktop\TableS2.xlsx#RANGE!_ENREF_1) |
| pCYR104-h9-lacZ1 | pCYR104 derivative, P_CI_-sglacZ1 with replaced handle h9 | [This study](file:///C:\Users\dai'er\Desktop\TableS2.xlsx#RANGE!_ENREF_1) |
| pCYR104-h10-lacZ1 | pCYR104 derivative, P_CI_-sglacZ1 with replaced handle h10 | [This study](file:///C:\Users\dai'er\Desktop\TableS2.xlsx#RANGE!_ENREF_1) |
| pCYR104-lacZ1-t1 | pCYR104 derivative, P_CI_-sglacZ1 with replaced terminator t1 | [This study](file:///C:\Users\dai'er\Desktop\TableS2.xlsx#RANGE!_ENREF_1) |
| pCYR104-lacZ1-t2 | pCYR104 derivative, P_CI_-sglacZ1 with replaced terminator t2 | [This study](file:///C:\Users\dai'er\Desktop\TableS2.xlsx#RANGE!_ENREF_1) |
| pCYR104-lacZ1-t3 | pCYR104 derivative, P_CI_-sglacZ1 with replaced terminator t3 | [This study](file:///C:\Users\dai'er\Desktop\TableS2.xlsx#RANGE!_ENREF_1) |
| pCYR104-lacZ1-t4 | pCYR104 derivative, P_CI_-sglacZ1 with replaced terminator t4 | [This study](file:///C:\Users\dai'er\Desktop\TableS2.xlsx#RANGE!_ENREF_1) |
| pCYR104-lacZ1-t5 | pCYR104 derivative, P_CI_-sglacZ1 with replaced terminator t5 | [This study](file:///C:\Users\dai'er\Desktop\TableS2.xlsx#RANGE!_ENREF_1) |
| pCYR104-lacZ1-t6 | pCYR104 derivative, P_CI_-sglacZ1 with replaced terminator t6 | [This study](file:///C:\Users\dai'er\Desktop\TableS2.xlsx#RANGE!_ENREF_1) |
| pCYR104-lacZ1-t7 | pCYR104 derivative, P_CI_-sglacZ1 with replaced terminator t7 | [This study](file:///C:\Users\dai'er\Desktop\TableS2.xlsx#RANGE!_ENREF_1) |
| pCYR104-lacZ1-t8 | pCYR104 derivative, P_CI_-sglacZ1 with replaced terminator t8 | [This study](file:///C:\Users\dai'er\Desktop\TableS2.xlsx#RANGE!_ENREF_1) |
| pCYR104-lacZ1-t9 | pCYR104 derivative, P_CI_-sglacZ1 with replaced terminator t9 | [This study](file:///C:\Users\dai'er\Desktop\TableS2.xlsx#RANGE!_ENREF_1) |
| pCYR104-lacZ1-t10 | pCYR104 derivative, P_CI_-sglacZ1 with replaced terminator t10 | [This study](file:///C:\Users\dai'er\Desktop\TableS2.xlsx#RANGE!_ENREF_1) |

^a)^ Km^R^ represent resistance to kanamycin. ^b)^ Specific multiplexed gRNA expression plasmids are not listed here. The gRNAs array is shown in Table S7.

**Table S5.** Sequence of P_J23119_-*rfp* as the reporter gene for one-pot assembly

| tctaga**gagacc**gttgacagctagctcagtcctaggtataatgctagccctaggaataattttgtttaactttaagaaggagatatacatatggcgagtagcgaagacgttatcaaagagttcatgcgtttcaaagttcgtatggaaggttccgttaacggtcacgagttcgaaatcgaaggtgaaggtgaaggtcgtccgtacgaaggtacccagaccgctaaactgaaagttaccaaaggtggtccgctgccgttcgcttgggacatcctgtccccgcagttccagtacggttccaaagcttacgttaaacacccggctgacatcccggactacctgaaactgtccttcccggaaggtttcaaatgggaacgtgttatgaacttcgaagacggtggtgttgttaccgttacccaggactcctccctgcaagacggtgagttcatctacaaagttaaactgcgtggtaccaacttcccgtccgacggtccggttatgcagaaaaaaaccatgggttgggaagcttccaccgaacgtatgtacccggaagacggtgctctgaaaggtgaaatcaaaatgcgtctgaaactgaaagacggtggtcactacgacgctgaagttaaaaccacctacatggctaaaaaaccggttcagctgccgggtgcttacaaaaccgacatcaaactggacatcacctcccacaacgaagactacaccatcgttgaacagtacgaacgtgctgaaggtcgtcactccaccggtgcttaaggaaggatccagacggatccaaa**ggtctc**ctcgct |
| --- |

**^a)^** BsaⅠ recognition sites are bolded. Sequence of *rfp* is shown in magenta.

**Table S6.** Primers used in this study

| **Primer** | **Sequence** |
| --- | --- |
| **Primers for amplification of separate sgRNA cassettes** | |
| CTAG-F | GCCGCGGGTCTCACTAGTAACACCGTGCGTGTTGACT |
| AGGT-R | GCCGCGGGTCTCAACCTAGTTCACCGACAAACAACAG |
| AGGT-F | GCCGCGGGTCTCAAGGTTAACACCGTGCGTGTTGACT |
| CCAC-R | GCCGCGGGTCTCAGTGGAGTTCACCGACAAACAACAG |
| CCAC-F | GCCGCGGGTCTCACCACTAACACCGTGCGTGTTGACT |
| GGTA-R | GCCGCGGGTCTCATACCAGTTCACCGACAAACAACAG |
| GGTA-F | GCCGCGGGTCTCAGGTATAACACCGTGCGTGTTGACT |
| TACG-R | GCCGCGGGTCTCACGTAAGTTCACCGACAAACAACAG |
| TACG-F | GCCGCGGGTCTCATACGTAACACCGTGCGTGTTGACT |
| TGAA-R | GCCGCGGGTCTCATTCAAGTTCACCGACAAACAACAG |
| TGAA-F | GCCGCGGGTCTCATGAATAACACCGTGCGTGTTGACT |
| ATCG-R | GCCGCGGGTCTCACGATAGTTCACCGACAAACAACAG |
| ATCG-F | GCCGCGGGTCTCAATCGTAACACCGTGCGTGTTGACT |
| GAAC-R | GCCGCGGGTCTCAGTTCAGTTCACCGACAAACAACAG |
| GAAC-F | GCCGCGGGTCTCAGAACTAACACCGTGCGTGTTGACT |
| TCGC-R | GCCGCGGGTCTCAGCGAAGTTCACCGACAAACAACAG |
| **Primers for genomic sites PCR amplification and sequencing** | |
| AampC-F | AGCCTTGGGTTAATTTTGCTTG |
| AampC-R | TCAGCCATTTTATTCGTTGGAA |
| AampD-F | CATGCTGGCGTATCTCGTTATG |
| AampD-R | CACAAGAATGGCGACCAGTAAT |
| AlacZ-F | TTACCCAACTTAATCGCCTTGC |
| AlacZ-R | TACCCGTAGGTAGTCACGCAAC |
| AtonB-F | TCAATTCACCGACCATTCA |
| AtonB-R | CAAGTCTCTACCGCCTCTA |
| AputB-F | GCCAAGGAGAAGCGATTC |
| AputB-R | TCTACCAGCCGTTCATCC |
| ApubA-F | GGCATAGGTCCATTCAATCT |
| ApubA-R | AATCTGGCGAGGTCATCT |
| AexbB-F | TAGAGGCGGTAGAGACTTG |
| AexbB-R | TGTGGAGCGTTAGGTGTT |
| AhmuA-F | TGATGAAGTGTTAGTGAGTG |
| AhmuA-R | GTCGTAATCTGTGCTATTCC |
| AputA-F | CGGCTCATGGAACAATCTGCG |
| AputA-R | GTTTTTGCCTTTGACGGGATCG |
| ApubC-F | GCCACTAACGCCTTATTACT |
| ApubC-R | AATCCGCATTCGCAAGTT |
| ApubB-F | CAGACGTTGTGCAGATTC |
| ApubB-R | GCGGCTTAACCTGATAATG |
| **Primers for pMBE sequencing to verify the insertion of gRNAs** | |
| SPME-F | CACCTCGCTAACGGATTCACC |
| SPME-R | TGCCCGCCAGTTGTTGTG |
| **Primers for RT-qPCR** | |
| gyrB-F | GGAACGACGGCTACCAAGA |
| gyrB-R | GTCAACGCACTACGGAAACC |
| sglacZ-F | GCGCAGCCTGAAGTTTT |
| sglacZ-R | CGACTCGGTGCCACTTTT |

**Table S7.** A set of orthogonal overhangs used to assembly multiple gRNAs

| AGGT |
| --- |
| CCAC |
| GGTA |
| TACG |
| TGAA |
| ATCG |
| GAAC |


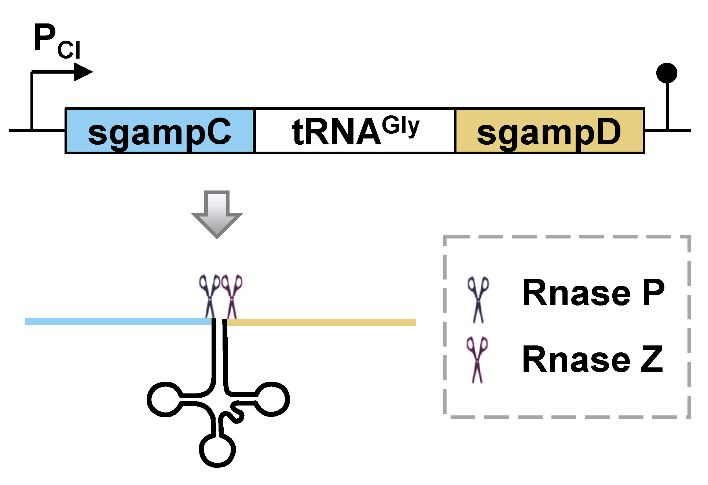


**Figure S1.** Schematic representation of the gRNAs processing of Design Ⅲ.


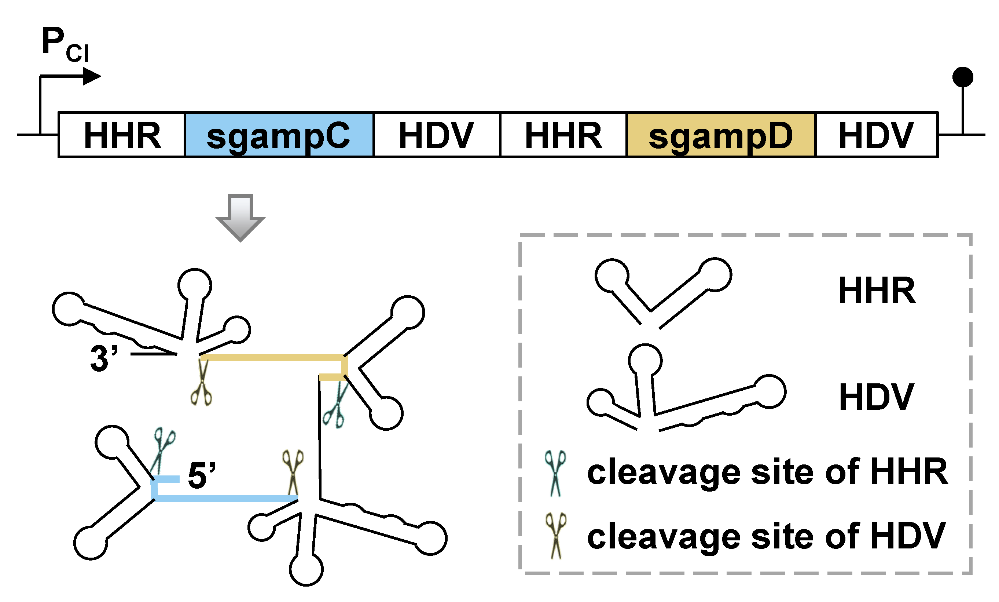


**Figure S2.** Schematic representation of the gRNAs processing of Design Ⅳ.


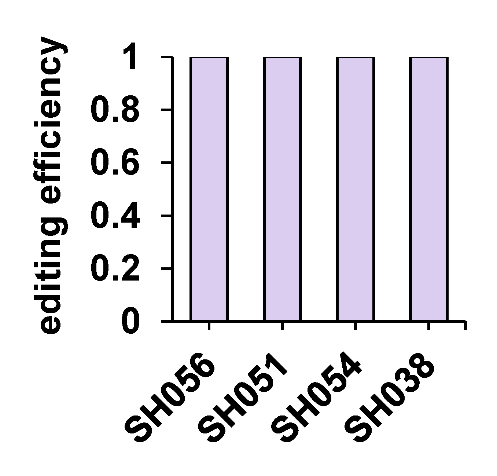


**Figure S3.** Editing efficiency of the gRNAs controlled by different promoters. Ten colonies were sequenced for each promoter.

**
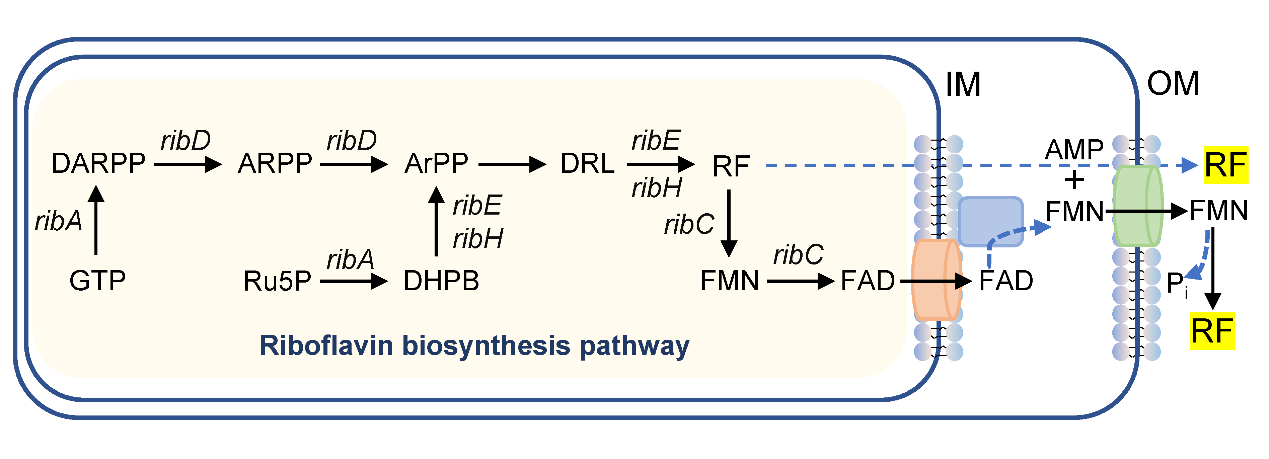
**

**Figure S4.** The riboflavin biosynthesis pathway. Abbreviation: Ru5P, Ribulose-5-phosphate; GTP, guanosine 5’-tri-phosphate; DARPP, 2,5-diamino-6-ribosyla-mino-4(3H)-pyrimidinone-5’-phosphate; ARPP, 5-amino-6-(5’-phosphoribosylamino)uracil; ArPP, 5-amino-6-(5’-phosphor-ibitylamino)uracil; DHPB, 3,4-dihydroxy-2-butanone 4-phosphate; DRL, 6,7-dimethyl-8-ribityl-lumazine; AMP, Adenosine monophosphate; FMN, flavin mononucleotide; FAD, flavin adenine dinucleotide; RF, riboflavin.


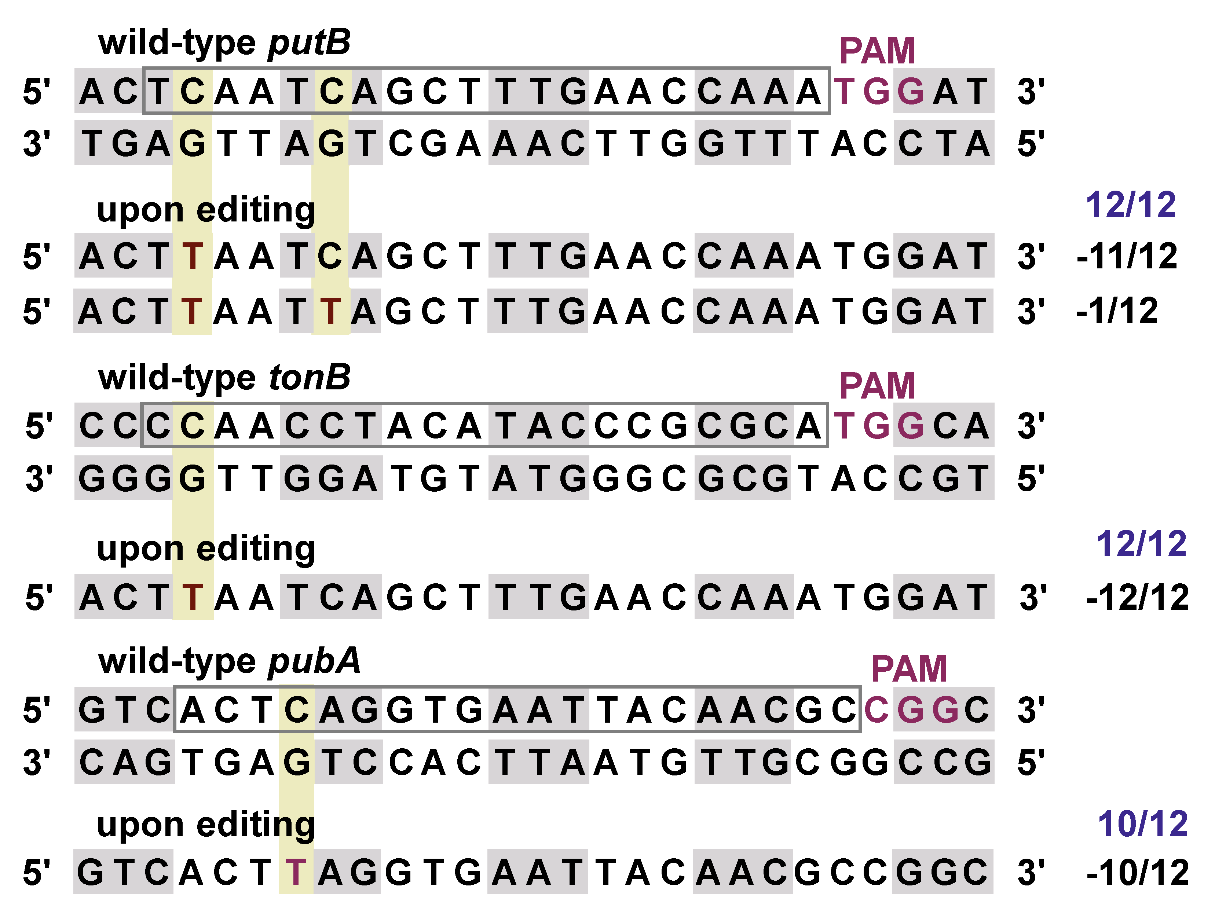


**Figure S5.** The editing sequence results of targeting 3 endogenous genes.


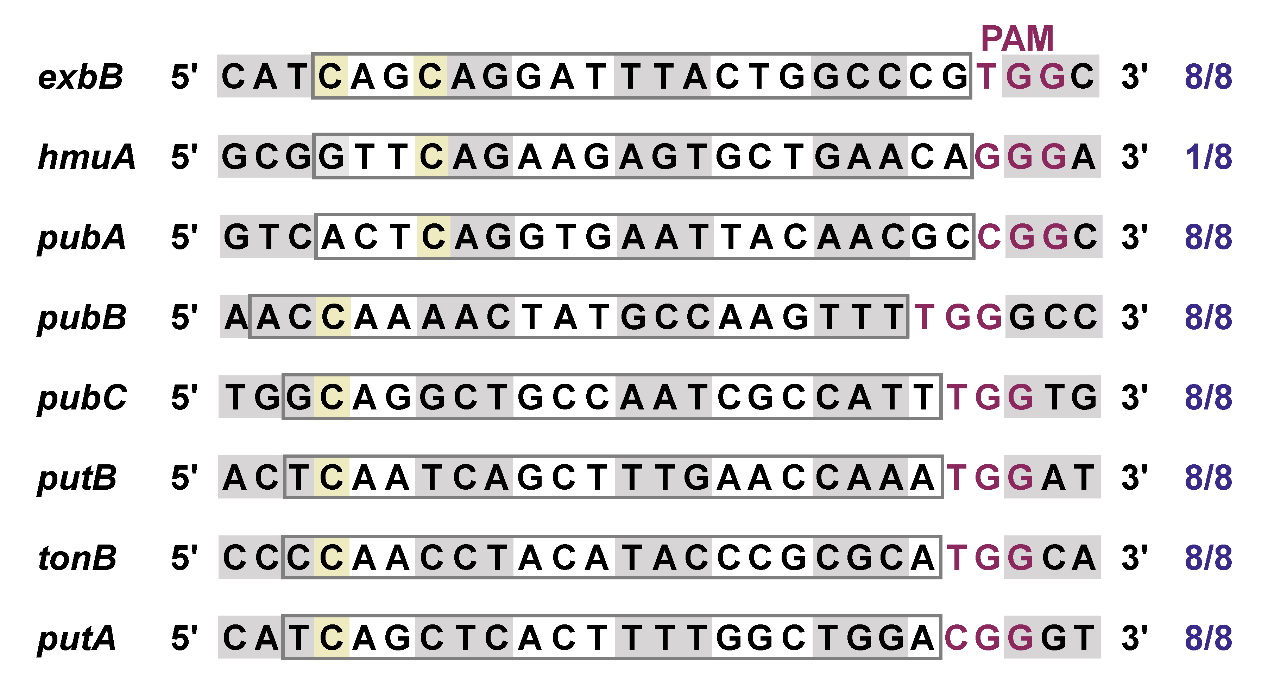


**Figure S6.** The editing sequence results of targeting 8 endogenous genes.


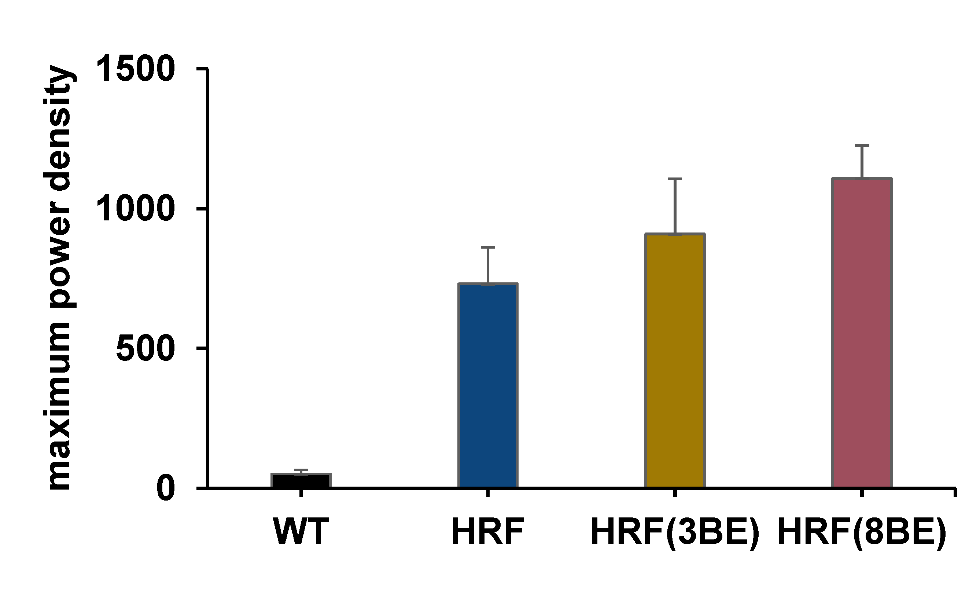


**Figure S7.** The maximum power density of multiplex engineered strains. Values and error bars indicate mean ± standard deviation of three replicates.

**Reference**

(1) Reis, A. C.; Halper, S. M.; Vezeau, G. E.; Cetnar, D. P.; Hossain, A.; Clauer, P. R.; Salis, H. M. Simultaneous repression of multiple bacterial genes using nonrepetitive extra-long sgRNA arrays. *Nat. Biotechnol.* **2019**, *37* (11), 1294-1301. DOI: 10.1038/s41587-019-0286-9.

(2) Corts, A. D.; Thomason, L. C.; Gill, R. T.; Gralnick, J. A. Efficient and Precise Genome Editing in Shewanella with Recombineering and CRISPR/Cas9-Mediated Counter-Selection. *ACS Synth. Biol.* **2019**, *8* (8), 1877-1889. DOI: 10.1021/acssynbio.9b00188.

(3) Corts, A. D.; Thomason, L. C.; Gill, R. T.; Gralnick, J. A. A new recombineering system for precise genome-editing in Shewanella oneidensis strain MR-1 using single-stranded oligonucleotides. *Sci. Rep.* **2019**, *9* (1), 39. DOI: 10.1038/s41598-018-37025-4.

(4) Yang, Y.; Ding, Y.; Hu, Y.; Cao, B.; Rice, S. A.; Kjelleberg, S.; Song, H. Enhancing Bidirectional Electron Transfer of Shewanella oneidensis by a Synthetic Flavin Pathway. *ACS Synth. Biol.* **2015**, *4* (7), 815-823. DOI: 10.1021/sb500331x.

(5) Chen, Y.; Fang, L.; Ying, X.; Cheng, M.; Wang, L.; Sun, P.; Zhang, Z.; Shi, L.; Cao, Y.; Song, H. Development of Whole Genome-Scale Base Editing Toolbox to Promote Efficiency of Extracellular Electron Transfer in Shewanella oneidensis MR-1. *Adv Biol (Weinh)* **2022**, *6* (3), e2101296. DOI: 10.1002/adbi.202101296.
